# Patient-ventilator synchrony in Neurally Adjusted Ventilatory Assist (NAVA) and Pressure Support Ventilation (PSV): a prospective observational study

**DOI:** 10.1186/s12871-015-0091-z

**Published:** 2015-08-08

**Authors:** Hodane Yonis, Laure Crognier, Jean-Marie Conil, Isabelle Serres, Antoine Rouget, Marie Virtos, Pierre Cougot, Vincent Minville, Olivier Fourcade, Bernard Georges

**Affiliations:** Service de Réanimation Polyvalente, CHU Rangueil, 1 Avenue Jean Poulhès, Pôle d’Anesthésie et Réanimation, TSA 50032, 31059 Toulouse, Cedex 9 France

**Keywords:** Neurally adjusted ventilatory assist, Pressure support ventilation, Intensive care unit, Electrical activity, Diaphragm

## Abstract

**Background:**

Weaning from mechanical ventilation is associated with the presence of asynchronies between the patient and the ventilator. The main objective of the present study was to demonstrate a decrease in the total number of patient-ventilator asynchronies in invasively ventilated patients for whom difficulty in weaning is expected by comparing neurally adjusted ventilatory assist (NAVA) and pressure support ventilation (PSV) ventilatory modes.

**Methods:**

We performed a prospective, non-randomized, non-interventional, single-center study. Thirty patients were included in the study. Each patient included in the study benefited in an unpredictable way from both modes of ventilation, NAVA or PSV. Patients were successively ventilated for 23 h in NAVA or in PSV, and then they were ventilated for another 23 h in the other mode. Demographic, biological and ventilatory data were collected during this period. The two modes of ventilatory support were compared using the non-parametric Wilcoxon test after checking for normal distribution by the Kolmogorov–Smirnov test. The groups were compared using the chi-square test.

**Results:**

The median level of support was 12.5 cmH_2_O (4–20 cmH_2_O) in PSV and 0.8 cmH_2_O/μvolts (0.2–3 cmH_2_O/μvolts) in NAVA. The total number of asynchronies per minute in NAVA was lower than that in PSV (0.46 *vs* 1, *p* < 0.001). The asynchrony index was also reduced in NAVA compared with PSV (1.73 vs 3.36, *p* < 0.001). In NAVA, the percentage of ineffective efforts (0.77 *vs* 0.94, *p* = 0.036) and the percentage of auto-triggering were lower compared with PSV (0.19 *vs* 0.71, *p* = 0.038). However, there was a higher percentage of double triggering in NAVA compared with PSV (0.76 *vs* 0.71, *p* = 0.046).

**Conclusion:**

The total number of asynchronies in NAVA is lower than that in PSV. This finding reflects improved patient-ventilator interaction in NAVA compared with the PSV mode, which is consistent with previous studies. Our study is the first to analyze patient-ventilator asynchronies in NAVA and PSV on such an important duration. The decrease in the number of asynchronies in NAVA is due to reduced ineffective efforts and auto-triggering.

## Background

Weaning from mechanical ventilation is typically performed with pressure support ventilation (PSV) [1]. This method allows for faster weaning in three quarters of patients who are invasively ventilated in the intensive care unit (ICU). However, 25 % of patients who have invasive ventilation have difficulty in weaning from mechanical ventilation. This is defined as failure of spontaneous breathing or resumption of mechanical ventilation within 48 h of removal [2]. For these patients, the time spent in weaning from mechanical ventilation can account for up to half of the total duration of invasive ventilation [3]. Most of these patients have chronic respiratory disease [4] (either chronic obstructive pulmonary disease or chronic respiratory failure), heart disease [3], or are undergoing long-term mechanical ventilation [2].

This difficulty in weaning is partly associated with the presence of asynchronies between the patient and the ventilator. Asynchronies affect approximately 25 % of the patients ventilated invasively and are responsible for an increase in duration of mechanical ventilation and increased length of stay in the ICU or hospital [5, 6]. In addition, the prolongation of duration of mechanical ventilation is a risk factor for occurrence of ventilator-associated pneumonia, which is a source of increased morbidity and mortality in the ICU [7].

The majority of asynchronies are represented by ineffective efforts, and/or double triggering [6]. However, asynchrony can be improved by optimum adjustment of ventilator settings (either a lower level of support, inspiratory trigger, expiratory cycling, setting up external positive external expiratory pressure [PEEP], or a combination of these). Despite optimal adjustments, asynchronies may persist and contribute to lengthening of the duration of weaning [8].

New modes of ventilation have been established in recent years to improve patient-ventilator synchrony [9]. Neurally adjusted ventilatory assist (NAVA) is one of these new modes. This mode is available in Servo-i® ventilators (Maquet Critical Care, Solna, Sweden) with the NAVA module. NAVA is an assisted ventilation, similar to PSV. NAVA uses an electromyographic signal of the diaphragm, which is filtered, processed, and integrated to obtain an electrical signal (EAdi) [9]. This electrical signal will trigger the ventilatory cycle, unlike the PSV mode, which relies upon detection of a difference in flow or pressure in the system to deliver the ventilator cycle. Collection of the electromyographic signal is performed by means of a nasogastric tube, with a multiple array of electrodes (EAdi catheter, Maquet Critical Care). Early detection of the patient’s inspiratory effort from the EAdi signal may reduce ineffective efforts [10, 11] and thus improve patient-ventilator interactions.

NAVA delivers proportional assistance. The level of pressure support varies from one cycle to the next cycle, and is proportional to the EAdi signal. The EAdi signal is proportional to the intensity of the diaphragmatic contraction. The more the diaphragmatic contraction is, the greater the level of support delivered by the ventilator is. However, if the support provided is too high, the nerve centers receive negative feedback, leading to detection of a difference in flow or pressure in the system from one cycle to the next, and therefore, there is less support. Moreover, if the diaphragmatic contraction is insufficient, positive feedback will cause a more powerful EAdi signal, and thus more support. This assistance allows proportional support to limit the periods of over- or under-assistance and provides the patient with more adaptation to physiological breathing [12]. If the EAdi signal is lost, this mode reverts to PSV.

This new mode of ventilation appears to be a promising method, especially in patients who present with difficulty weaning. In recent years, several studies [10, 11, 13–15] have shown a decrease in the number of patient-ventilator asynchronies in NAVA compared with PSV. All of these studies only analyzed periods of 10 to 30 min and, in most of them, PSV was not optimized, especially regarding the expiratory cycling. One study that included only surgical patients showed the stability of the EAdi for periods of 24 h [16]. Nevertheless, this study did not examine patient-ventilator asynchrony, but investigated criteria of oxygenation and variability of the ventilatory parameters. Another study investigated the NAVA level of titration taking into account the level of pressure support of 7 cmH_2_O and a PEEP level of 0 cmH_2_O [17]. This previous study aimed to titrate the level of assistance in NAVA and confirm implementation of the method on ventilator weaning.

To the best of our knowledge, no clinical study has analyzed the number of asynchronies in the NAVA mode and compared it in PSV over 23 h in patients receiving invasive ventilation over a long period or with comorbidities. Therefore, the main objective of the present study was to determine if there is a decrease in the total number of patient-ventilator asynchronies in invasively ventilated patients for whom difficulty in weaning is expected in NAVA compared with PSV. The secondary objectives were to determine if there is a decrease in different types of asynchronies (ineffective efforts, auto-triggering, and double triggering), a better respect of a “setpoint” of tidal volume (VT) between 6 and 8 ml/kg of predicted body weight (PBW), and a greater variability of VT in NAVA compared with PSV.

## Methods

We conducted a prospective, non-randomized, non-interventional, single-center study. This was an observational study, which was approved by the Ethics Research Committee of the University Hospital of Toulouse (France) (number 14–0312). Before inclusion, patients and/or families gave their consent about participation after information on the aim of this study.

The study was conducted in the service of the ICU of Rangueil Hospital over a period of 12 months. Inclusion criteria for the study were that patients had to be invasively ventilated and present with predictive criteria of difficult weaning [2]. Difficult weaning was defined as a high duration of mechanical ventilation, or a history of respiratory (chronic obstructive pulmonary disease and restrictive disease), heart (left heart failure and coronary artery disease) or neuromuscular diseases. Exclusion criteria included the following: contraindication to EAdi catheter placement (e.g., recent gastric or esophageal surgery and the presence of esophageal varicose veins); presence of a tracheotomy; a progressive infectious process, such as nosocomial pneumonia, which was defined with at least two of the following criteria: rectal temperature > 38.5 °C or < 36.5 °C, mucopurulent bronchial secretions, recent or persistent diffuse or localized parenchymatous infiltrate on pulmonary X-ray, and hyperleukocytosis greater than 12 G/L or leukopenia less than 5 G/L, associated with a positive bacteriological swab obtained by bronchoalveolar washing (positive if ≥ 10^4^ CFU/ml) or by tracheal aspiration (positive if ≥ 10^6^ CFU/ml); nosocomial bacteremia, defined in accordance with the Bone criteria for a septic syndrome; hemodynamic failure with a mean arterial pressure less than 65 mmHg or a need for catecholaminergic treatment; decision to withhold life-sustaining treatment; and presence of a guardianship.

**Fig. 1:**
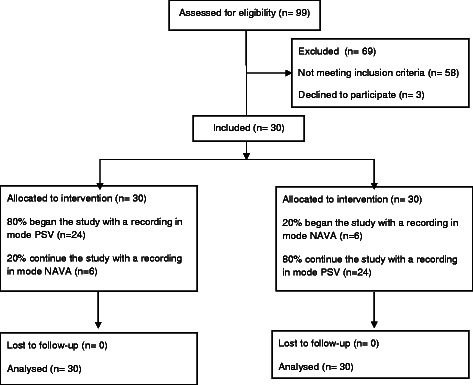
Inclusion of the patients

Patients were able to be included in the study when sedation was stopped and the patients met the general and respiratory criteria for being in PSV [2] (Fig. 1). Included patients benefited from placement of the EAdi catheter.

Patients were then successively ventilated for 23 h using NAVA or PSV and then they were ventilated for another 23 h in the other mode. The patient’s physician optimized the ventilator settings in the particular PSV mode to limit the appearance of asynchronies. Within the team, only three physicians optimized the settings of the ventilation according to the aim of the protocol.

For PSV, before collecting data, we adjusted the inspiratory trigger in-flow, the level of support to obtain a VT between 6 and 8 ml/kg of PBW, and the external PEEP, and adapted to the level of intrinsic PEEP and expiratory cycling. The inspiratory trigger and expiratory trigger were kept constant during recording, while the level of support was adapted to the VT during the nychthemeron. These settings of PSV were the same for those who started with NAVA. These patients initially had PSV, but recording began with the NAVA mode. In NAVA, we used one of the functions of the ventilator Servo-i® (preview NAVA) to estimate the NAVA gain to obtain the same peak pressure as during PSV. This NAVA gain was mostly unchanged during recording. The EAdi inspiratory trigger was set to a predetermined default value of 0.5 μvolts, always above the minimal value of EAdi of the patient. The cycle-off value was fixed at 70 % of peak EAdi in the NAVA mode. PEEP in NAVA was left at the same level as PEEP in PSV. NAVA includes a safety feature whereby, in case the Eadi signal has artifacts or is lost, the ventilator automatically reverts to PSV.

### Data collection

**Fig. 2:**
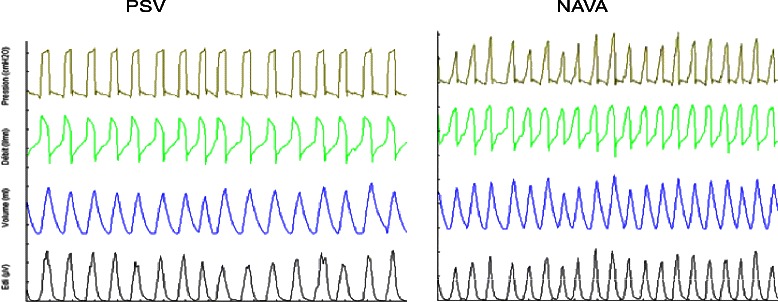
Examples of recording data in PSV and NAVA

To collect data from the ventilator, we used software (Servo-i software CPR, Maquet Critical Care) to determine respiratory curves during recovery. We recorded pressure curves, flow curves, volume curves, and diaphragmatic signals (EAdi) (Fig. 2). We then proceeded to collection of VT and levels of assistance on a paper chart record.

### Data analysis

Two independent senior experts analyzed the data recorded by the software in pairs. In case of disagreement within the pair, a third physician was requested. Analysis of respiratory curves was manually performed by analyzing the first 5 min of recording every 4 h, which represented an analysis period of 25 min.

**Fig. 3:**
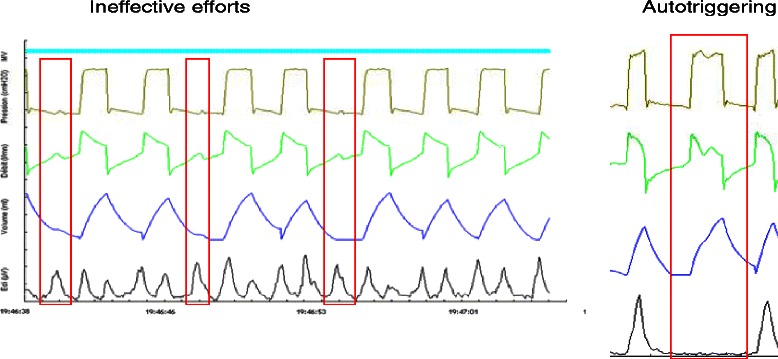
Ineffective efforts and auto-triggering

**Fig. 4:**
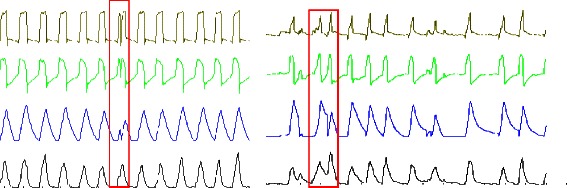
Double triggering

Three types of asynchronies were analyzed: (1) ineffective efforts defined by the existence of an EAdi signal, without a ventilator cycle (Fig. 3); (2) auto-triggering defined by the presence of a ventilator cycle without a diaphragmatic signal (Fig. 3); and (3) double triggering, which was defined by the presence of two successive cycles without intermediate expiration or an interrupted exhalation, or by a biphasic aspect of the EAdi signal, which leads to two successive machine cycles (Fig. 4). The total number of asynchronies was then calculated for each ventilation mode by adding different asynchronies recorded during the first 5 min of each period of 4 h and for 23 h of recording.

The asynchrony index (AI) was calculated. This index corresponds to the total number of asynchrony events/numbers of EAdi signals × 100. The AI has been used in other studies [13, 14]. For each type of asynchrony, we calculated the percentage of asynchronies as follows: number of each asynchrony events / total number of cycles over the period analyzed × 100. In addition, we calculated the VT in ml/kg of PBW, corresponding to the different recording periods. The variability of VT in each ventilation mode was evaluated by the coefficient of variation. The characteristics of the population and the variables are shown as mean ± standard deviation, median, and interquartile. The two modes of ventilatory support were compared using the non-parametric Wilcoxon test after determining normal distribution by the Kolmogorov–Smirnov test. Comparison of the groups was performed by the chi-square test (with Yate’s correction when necessary). Analyses were performed using Statview 5.0 software. Differences between groups were considered statistically significant for values of *p* < 0.05. The main objective was the difference in the total number of patient-ventilator asynchronies in NAVA compared with PSV. Power analysis indicated that a sample size of 28 was sufficient to demonstrate a 20 % reduction in the number of asynchronies between PSV and NAVA modes, with α and β risks of 0.05 and 0.20 respectively.

## Results

### Population

A total of 30 patients who were invasively ventilated in PSV, without sedation, and had risk factors for difficult weaning were included (Table 1). Of these 30 patients, three were trauma patients, 14 were medical patients, and 13 were surgical patients. A total of 56.6 % of our patients had a known respiratory disease and 36.6 % had a known cardiac disease. A total of 33 % of patients were tracheotomized after our study for difficult weaning. To be enrolled in the study, the initial pathology in the patients was stabilized, and they were weaned with the PSV or NAVA mode.

**Table 1:** Characteristics of the population

Age (years)	66.3 ± 11
M/F	19/11
BMI (kg/m^2^)	28.9 ± 6.6
SAPS II	58.6 ± 20.7
Known pulmonary disease	17 (56.6 %)
Known heart disease	11 (36.6 %)
ARDS	15 (50 %)
Post study tracheotomy	10 (33 %)
Duration of ventilatory support (days)	32.8 ± 21.6
ICU LOS (days)	35.9 ± 21
Mortality at day 28	5 (16.7 %)
Overall mortality	10 (33.3 %)

Data are presented as mean ± SD or number (%)

The total duration of the ventilation was 32 ± 21 days. The mortality at day 28 was 16.7 % and the overall mortality was relatively high at 33.3 % during the hospital stay.

There was no significant difference in Simplified Acute Physiology Score II for the three subgroups of patients (trauma, medical, or surgical). Table 2 shows the main characteristics of these patients. Twenty-four patients, which represented 80 % of our population, began the study with recording in the PSV mode, which is the most commonly used in the unit.

**Table 2:** Main ventilator settings

	PSV	NAVA	*P*
Level of support	12.5 [4–20] cmH2O	0.8 [0.2–3] cmH2O/μvolt	
Inspiratory trigger	flow	-	
Expiratory trigger (%)	30 [21–40]	-	
Asynchrony			
- number (events/min)	1 [0–17]	0.46 [0.08–7.84]	0.0006*
-AI (%)	3..37 [0–34.47]	1.73 [0.24–20]	0.0015*
- number of patients with an AI > 10 %	9/30 (30 %)	5/30 (16.6 %)	0.0013*
Ineffective efforts			
- number (events/min)	0.22 [0–16.96]	0.22 [0–7.64]	0.0259*
- percentage (%)	0.94 [0–34.4]	0.77 [0–17.73]	0.0369*
Autotriggering			
- number (events/min)	0.18 [0–2.76]	0.04 [0–0.48]	0.0100*
- percentage (%)	0.71 [0–9.54]	0.19 [0–2.94]	0.0385*
Double triggering			
- number (events/min)	0.1 [0–4.72]	0.20 [0–4.76]	0.036*
- percentage (%)	0.71 [0–9.54]	0.76 [0–18.9]	0.046*
VT			
- ml/Kg of PBW	6.6 [4.3–12.2]	6.7 [5.2–10.6]	0.48
- percentage of patients with a VT between 6 and 8 mlkg of PBW	43.3	60	0.016*
- Variability	0.12 [0.01–0.41]	0.13 [0.02–0.91]	0.18
- Variability > 13 % (%)	33	53	0.04*

Data are presented as median [interquartile range]. * is signicant with *p* lower than 0.05

### Ventilation parameters

The results are shown as median and range. The median level of support was 12.5 cmH_2_O (4–20 cmH_2_O) in PSV and 0.8 cmH_2_O/μvolts (0.2–3 cmH_2_O/μvolts) in NAVA. The expiratory trigger was 30 % (21–40 %) in PSV. The total number of asynchronies per minute in NAVA was lower than that in PSV (0.46 *vs* 1, *p* = 0.0006). The asynchrony index in NAVA was also lower compared with PSV (1.73 *vs* 3.36, *p* = 0.0015). The number of patients with an AI > 10 % was lower in NAVA than in PSV (16.6 % *vs* 30 %, *p* = 0.0013).

**Fig. 5:**
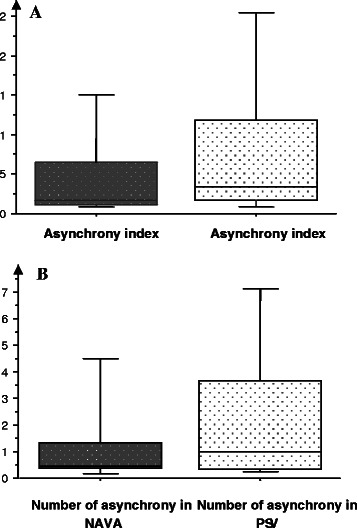
AI (**a**) and number (**b**) of asynchronies in NAVA and PSV

In NAVA, the percentage of ineffective efforts (0.77 *vs* 0.94, *p* = 0.036) and the percentage of auto-triggering (0.19 *vs* 0.71; *p* = 0.038) were lower than those in PSV. However, there was a higher percentage of double triggering in NAVA compared with PSV (0.76 *vs* 0.71, *p* = 0.046, Fig. 5). When we analyzed asynchronies in the same patient and compared NAVA with PSV, we observed that in 22 (73 %) patients, the AI in the NAVA mode was lower than that in PSV.

There was no difference in VT between NAVA and PSV (6.7 *vs* 6.6, *p* = 0.48). However, a higher number of patients on NAVA (60.0 %) had a VT between 6 and 8 ml/kg of PBW than those on PSV (43.3 %, *p* = 0.016). There was no significant difference in the variability in VT between NAVA and PSV. However, when taking the median as the reference of variability up to 13 %, we found that the number of patients with high variability was higher for NAVA than for PSV (53 % vs. 33 %, *p* = 0.04).

### Oxygenation parameters

Table 3 shows arterial blood gases and hemodynamics during PSV and NAVA. PaO_2_ and PaO_2_/FiO_2_ were significantly higher during the NAVA mode compared with PSV (both *p* < 0.0001).

**Table 3:** Arterial blood gases and hemodynamics during PSV and NAVA

	PSV	NAVA	*P*
pH	7.43 [7.24–7.57]	7.43 [7.25–7.51]	0.3011
PaO_2_ (mmHg)	66.7 [54.6–116.8]	77.4 [61.4–115.4]	0.0001*
PaO_2_ / FiO_2_	203.15 [113.2–389.3]	254.3 [136.4–409.6]	<0.0001*
Pa CO_2_ (mmHg)	41.5 [27.2–68.4]	40.9 [27.3–61.1]	0.4839
HCO3- (mmol/L)	27.4 [18.3–39.3]	27.2 [15.8–37.2]	0.1229
MAP (mmHg)	82 [66–102]	79.5 [65–100]	0.402
HR (beats/min)	94 [51–129]	91 [62–128]	0.234

Data are presented as mean ± SD or median [interquartile range]. * is signicant with p lower than 0.05

## Discussion

Our study showed that a lower total number of asynchronies in NAVA compared with PSV reflected an improved patient-ventilator interaction. This finding is consistent with results of previous studies [10, 11, 13–15]. This reduction in the number of asynchronies is related to a reduction in ineffective efforts and auto-triggering. Ineffective efforts could be due to the presence of intrinsic PEEP, which increases the patient’s effort required to trigger the ventilator. Ineffective efforts could also be due to excessive levels of pressure. The consequences of ineffective efforts are that the patient’s inspiratory effort will fail to trigger a ventilator breath [9]. In our study, we attempted to optimize the pressure level and the expiratory trigger in the PSV mode. Unlike other studies [10, 11, 13, 15], our study showed persistence of ineffective efforts in NAVA. This finding could be related to the fact that the trigger in NAVA relies on the principle “first detected, first served”.

The ventilator initiates the machine cycle according to the first trigger it has detected. This trigger is a pneumatic trigger or a trigger based on the EAdi signal. In our study including 56.6 % of patients with a known respiratory disease, we observed that some patients used their accessory breathing muscles to trigger the ventilator. Therefore, a pneumatic trigger was rewarded before onset of the diaphragmatic signal. The machine cycle beginning before the EAdi signal, and having no way to detect if the data stored is a pneumatic trigger or trigger signal based on the Eadi. We could analyze some cycles triggered by a pneumatic trigger as asynchronous cycles (auto-triggering followed by ineffective efforts). Another possible explanation for this auto-triggering is that we took into account all of the diaphragmatic signals in our analysis. However, some signals may correspond to artifacts (cardiac activity). Mauri et al. [13] excluded some diaphragmatic signals, considering them as artifacts, in their study.

In our study, double triggering was more frequent in NAVA than in PSV. This finding is consistent with the results of Piquilloud and colleagues [10]. This larger number of double triggering in NAVA is related to the fact that sometimes there are EAdi signals with a biphasic appearance, and this causes two successive cycles (Fig. 3). This biphasic appearance could be related to early cycling when the inspiratory time of the ventilator is less than the neural inspiratory time of the patient. This may not increase the work of breathing, but it may participate in the discomfort felt [10].

In our study, the NAVA level was not optimized, which could have affected the persistence of asynchronies. The initial setting of the NAVA gain was based on the level of assistance in PSV. In most other studies [10, 11, 14, 15], the NAVA gain was set in the same way as in our study using the “NAVA preview” function of the ventilator Servo-I, and this technique is recommended by the manufacturer. In addition, the NAVA gain was not changed much during the nychthemeron, unlike the level of assistance in PSV. This finding is probably due to less control of this new type of ventilation compared with PSV, which is the reference mode.

A better way to settle the NAVA level might be by using the method of Roze and colleagues [17]. Their method is based on daily titration of the NAVA level according to the maximum EAdi signal that is obtained during a spontaneous breathing trial with a level of support of 7 cmH_2_O and a PEEP level of 0 cmH_2_O. We unexpectedly found a relatively low AI in NAVA and PSV. This finding can be explained by the fact that all of our settings, at least in PSV, were optimized with particular attention to setting the level of assistance and expiratory cycling depending on the condition of the patients.

To the best of our knowledge, this is the first study to compare asynchronies in PSV and NAVA where ventilator settings in PSV have been optimized. However, we did not analyze other types of patient-ventilator asynchronies, such as early or late cycling, which could artificially underestimate the total number of asynchronies, and thus the AI. Thille and colleagues [6] showed that an AI greater than 10 % is associated with an increase in the duration of mechanical ventilation and an increase in use of tracheotomy for ventilator weaning. In our study, less patients using NAVA than those using PSV had an asynchrony index greater than 10 %. NAVA could be helpful in patients with difficult weaning by reducing the number of asynchronies, particularly in patients with a high AI.

We did not find any difference in VT between NAVA and PSV, similar to most previous studies [10, 13, 15]. However, with NAVA, more patients had a VT between 6 and 8 ml/kg of PBW than those with PSV. This finding suggests that in NAVA, over 23 h, periods of over- and under-assistance are relatively limited compared with PSV.

This setting of VT between 6 and 8 ml/kg is recommended in protective ventilation in acute respiratory distress syndrome to reduce the risk of barotrauma and volutrauma [18]. However, some studies appear to suggest that this protective ventilation may also reduce the risk of ventilator-induced lung injury in patients without acute respiratory distress syndrome and those who are ventilated invasively [19, 20]. Several studies have demonstrated a reduced risk of over-assistance in NAVA compared with PSV [14, 15, 21]. In contrast, few studies have focused on the risk of under-assistance.

In a previous study of postoperative patients with thoracic or abdominal surgery, a few patients had a VT less than 5 ml/kg, with no signs of discomfort or respiratory distress [16]. However, the setting of VT is normally between 6 and 8 ml/kg of PBW, and a lower level of VT should not be used only on the basis of this previous study. However, we can assume that for some patients with good clinical and biological tolerance, a lower VT can be accepted. In NAVA, the majority of previous studies found variability in VT, and this is one of the benefits, at least theoretically, of proportional ventilation modes [12, 15, 16, 22]. In our study, we do not find this variability in VT. This lack of finding is probably due to the small size of our population. In addition, when recording VT on paper, at a given moment, this variability might be hidden. However, we also examined the number of patients who had variation in VT of more than 13 %, which corresponds to the median of the variability of VT in our series. We found that more patients in NAVA had variability of VT greater than the median of variability than those in PSV.

In contrast to Piquilloud et al’s study [10], but in agreement with Terzi et al’s study [9], we found improvement in the parameters of oxygenation in NAVA. These findings could have resulted from better patient-ventilator synchronization and a more natural breathing pattern, which may also contribute to improved gas exchange [9].

Our study has some limitations. First, the absence of randomization could have introduced bias in the study in terms of adaptation to the ventilatory mode, but the study aim was not to compare the duration of weaning. Second, not analyzing early or late cycling could have led to underestimation of the total number of asynchronies, and thus the AI. Early or late cycling is observed with the PSV mode, but not with the NAVA mode [10]. Despite recording for 23 h, analysis of respiratory curves was manually analyzed for the first 5 min of recording every 4 h, which represents an analysis period of 25 min.

Finally, our study, which recorded data for 23 h, similar to Coisel and colleagues’ study [16], confirms the stability of the EAdi signal. This appears to be the primum movens before considering this new ventilation mode as a potential mode of weaning from mechanical ventilation.

## Conclusion

To the best of our knowledge, this is the first study to focus on patient-ventilator asynchronies in NAVA and PSV in patients with difficult weaning criteria and for such a long period. There are fewer asynchronies in NAVA, with reduced ineffective efforts and auto-triggering compared with PSV. NAVA also reduces the risk of over- and under-support, while providing more physiological ventilation with VT variability than PSV. Further studies are required to determine the clinical impact of this improved synchrony.

## Abbreviations

NAVA: Neurally adjusted ventilatory assist
PSV: Pressure support ventilation
ICU: Intensive care unit
EAdi: Electrical activity of the diaphragm
VT: Tidal volume
PBW: Predicted body weight
PEEP: Positive external expiratory pressure
AI: Asynchrony index
FiO: Inspiratory oxygen fraction
